# Evolution of the RNA Cleavage Subunit C11/RPC10, and Recycling by RNA Polymerase III

**DOI:** 10.33696/immunology.4.133

**Published:** 2022

**Authors:** Saurabh Mishra, Richard J. Maraia

**Affiliations:** 1 Department of Biochemistry, Institute of Science, Banaras Hindu University, Varanasi, India; 2 Intramural Research Program of the Eunice Kennedy Shriver National Institute of Child Health and Human Development, National Institutes of Health, Bethesda, MD USA

**Keywords:** RNA:DNA hybrid, RNA-3’ cleavage, Transcription elongation, Transcription termination, Reinitiation-recycling, RNA polymerase III, C11, C37/53 heterodimer, RPC10, TFIIS

## Abstract

Nuclear RNA polymerase (Pol) III synthesizes large amounts of tRNAs and other short non-coding (nc)RNAs by a unique process that involves a termination-associated reinitiation-recycling mechanism. In addition to its two largest of 17 subunits, which contribute to active center RNA-DNA binding and catalytic site, a smaller subunit of ~110 aa (yeast C11, human RPC10) monitors this site, can modify its activity, and is essential for reinitiation-recycling. Distinct, but relevant to human immunity is cytoplasmic (cyto-)Pol III that is a direct sensor of AT-rich viral DNA from which it synthesizes 5’-ppp-RNA signaling molecules that activate interferon (IFN) production. Mutations in genes encoding Pol III subunits cause severe anti-viral immunodeficiency although the mechanisms responsible for cyto-Pol III initiation on this AT-rich DNA are unknown. Cyto-Pol III has also been implicated in inducing IFN in response to cytosolic mitochondrial DNA in autoimmune dysfunction. A focus of this commentary is recent biochemical and genetics research that examined the roles of the individual domains of C11 in the Pol III termination-associated reinitiation-recycling process as well as more recent cryo-EM structural and accompanying analyses, that are considered in evolutionary and other biological contexts. The N-terminal domain (NTD) of C11/RPC10 anchors at the periphery of Pol III from which a highly conserved linker extends to the mobile C-terminal RNA cleavage domain that can reach into the active center and rescue arrested complexes. Biochemical data indicate separable activities for the NTD and CTD in the transcription cycle, whereas the NTD-Linker can confer the evolutionary unique Pol III termination-reinitiation-recycling activity. A model produced from single particle cryo-EM conformations indicates that the C11-Linker-CTD swings in and out of the active center coordinated with allosteric movements of the DNA-binding clamp by the largest subunit, coupling termination to reinitiation-recycling. These may be relevant to DNA loading by cyto-Pol III during immune signaling.

## Introduction

All cellular RNAs are synthesized by evolutionary related DNA-dependent multisubunit RNA polymerases (Pols). Bacteria and archaea each use a single Pol to synthesize all their RNAs whereas a hallmark of eukaryotes is three homologous Pols, I, II and III and the associated Pol-specific transcription factors (TFs) to regulate synthesis of different class RNAs [1,2]. The Pol III system produces high molar amounts of tRNAs and other small ncRNAs by the efficient reuse of its stable transcription complexes after formation on the promoters of its target genes, and the recycling of Pol III itself [1].

Advances using human cell and budding yeast *S. cerevisiae* components revealed that the 17-subunit Pol III enzyme, its two general multisubunit TFs, TFIIIB and TFIIIC, the 5S-specific TFIIIA, and general repressor, Maf1, are highly conserved [3-5]. Early studies established basic mechanisms involved in transcription initiation including some that are shared or very similar in the Pols II and I systems [2].

While the internal promoters and transcription mechanisms used for the major Pol III-dependent genes, the tRNAs have been conserved, one of the 17 conserved subunits of Pol III itself, RPC5 was massively enlarged during eukaryotic evolution by acquisition of new domains [1,6,7]. In addition, the Pol III type-3 promoter and its multisubunit TF, SNAPc appeared in higher eukaryotes, and many new Pol III-dependent ncRNA genes appeared, some of which function in cellular immunity [1, and refs therein].

Thus the fact that the core of the Pol III system is conserved from yeast to human should not overshadow key connections that arose between its regulation and some biological processes that central to higher eukaryotes such as cellular differentiation, development, and immunity [1, and refs therein]. It is especially notable for this review that a radically new activity for Pol III adapted to vertebrates. Viral AT-rich DNA is specifically detected by cytoplasmic-localized Pol III (cyto-Pol III), which was apparently an evolved activity as an innate immune sensor in one of the several activation pathways for type I and III interferons (IFNs) [8,9]. In this function cyto-Pol III appears to use a promoter-*in*dependent mechanism of transcription initiation to produce the 5’ppp-RNA signaling molecules from the AT-rich DNA [critically reviewed in 1] that bind and activate retinoic acid-inducible gene I (RIG-I) through the mitochondrial antiviral-signaling protein (MAVS) and TBK1 (TANK-binding kinase-1) pathway [10]. We note that the mechanism used by cyto-Pol III for this mode of initiation, which is presumably transcription factor-*in*dependent, is unknown [critically reviewed in 1]. Heterozygous mutations in genes encoding four Pol III subunits, *POLR3A*/RPC1, *POLR3C*/RPC3, *POLR3E*/RPC5, *POLR3F*/RPC6, compromise this immune response to Varicella Zoster Virus (chickenpox) [10]. A minority of the eleven immunodeficient *POLR3* alleles are in the core subunit, *POLR3A*/RPC1, whereas the majority are colllectively in the initiation subunits POLR3C, POLR3F, and the termination-reinitiation subunit POLR3E (Table 1) [10]. Cyto-Pol III has also been implicated in type I IFN response to cytosolic mitochondrial (mt)DNA potentially involved in autoimmune disease such as systemic lupus erythematosus [11,12].

**Figure 1:**
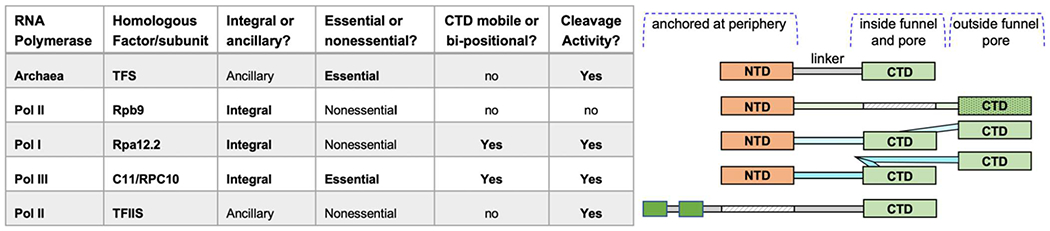
Phylogenetic-evolutionary relationships of NTD and CTD of the Rpb9, Rpa12.2, C11/RPC10, TFIIS and TFS homologous factors/subunits and their associated RNA polymerases. Associated features and characteristics discussed in the text are listed on the left side of the figure. Rpa12.2 and C11/RPC10 each have only one CTD that is depicted in the two alternative positions discussed in the text. The linkers are shown as solid thin rectangles; hatched rectangles represent absence of domain. The N-terminal region of TFIIS is unrelated to the others. The figure was inspired by [28].

We shall now discuss recent developments involving the intrinsic transcript cleavage subunit of Pol III, C11/RPC10. As is illustrated in Figure 1, C11/RPC10 is a sequence homolog of the Pol II integral subunit Rpb9 but is distinguished by CTD that is both mobile and active for transcript cleavage. C11/RPC10 is also integral to the process of termination-associated reinitiation-recycling of Pol III onto preassembled transcription complexes. In this process, which is unique to Pol III, C11 is dependent on its associated heterodimer subunits, yC37-C53. Although Pol III can autonomously terminate transcription upon synthesizing RNAs that contain a short 3’ oligo(U) sequence that reflect the oligo(dT) termination signal in the non-template (NT) DNA [13,14] (i.e., oligo-A on the template strand) multiple studies have documented specific positive effects of the C37/53 heterodimer on promoting the efficiency of this termination process [14-19].

The focus of recent work by Mishra et al. was to examine the roles of the three domains of C11, the NTD, Linker and CTD, in Pol III termination-associated reinitiation-recycling and related activities *in vitro*, and contributions to the essential *in vivo* cell viability function of C11 [18]. A surprising result was that the essential function of C11 does not require its CTD-mediated transcript cleavage activity, nor the presence of the CTD. Consistent with this, CTD-mediated transcript cleavage activity, nor the presence of the CTD were required for termination or reinitiation-recycling by Pol III *in vitro* [18]. The C11 NTD connected to the Linker (NTD-L) was found to confer the essential function of C11 *in vivo*, and separately, Pol III termination-associated reinitiation-recycling activity of C11 *in vitro* [18].

The C37/53 heterodimer can increase termination efficiency by C37/53 without C11, whereas its ability and requirement to promote reinitiation-recycling by Pol III requires C11 [15,18]. Consistent with these data, two recent cryo-EM analyses, of human (h)Pol III and *S. cerevisiae* (sc)Pol III led to a newly described conserved exit tunnel for the NT-DNA and evidence for oligo-dT as a length-dependent pause element that comprises a pre-termination complex (PTC). A conserved network of RPC2-specific hydrogen bonds form with oligo-dT. Moreover, contraction of the RPC2 lobe region which accommodates sequential dT-specific methyl groups in a hydrophobic pocket, traps the termination sequence [19,20]. Additional analysis indicate that a C-terminal region of C37 known to prevent terminator readthrough (RT), acts indirectly by stabilizing the trap around the oligo-dT sequence, which would enhance the pause [19,20].

Elongating RNA polymerases pause at certain DNA sequences which can assist proper RNA folding [reviewed in 21,22], or may proceed to a pre-termination complex [23]. Long pauses may generally lead to arrest with detachment of the RNA 3’ end from the catalytic site while the active center maintains grip on the RNA-DNA hybrid. Arrest can result from multiple causes, and is potentially devastating to genome integrity and to the organism [24]. An important RNA polymerase response to arrest that is conserved from bacteria to eukaryotes is backward movement on the template, termed backtracking, during which the RNA 3’ end is extruded from the active site into a secondary channel (not the main DNA binding cleft) used for NTP entry, hereafter referred to as the funnel [25,26]. A next step in the arrest rescue response is active site remodeling for intrinsic transcript cleavage [27,28]. While polymerase active sites can exhibit transcript cleavage it is limited to low level intrinsic activity, but they can recruit factors with catalytic site remodeling activity that greatly enhance it [28,29]. Some of these factors have been shown to enter the funnel and position their catalytic residues to realign the active site to cleave the RNA as an endoribonuclease. This creates a new RNA-3’OH end that is precisely positioned for elongation [see cartoon model Figure 4 in ref. 25]. It was proposed that such activity of C11/RPC10 may also facilitate and/or rescue termination at a fraction of terminators at which Pol III may arrest [18].

### Mobility of C11/RPC10 is critical for Pol III termination-reinitiation-recycling

Studies of hPol III in ECs that included examination of allosteric changes derived from “3D analysis” of many single cryo-EM particles, as well as biochemical examination of different phases of *in vitro* transcription reactions, led to similar models [7,18] in which mobility of either the C11 NTD-Linker alone or including the CTD is critical for termination-associated reinitiation by Pol III [7,18] [also see 19]. Cryo-EM of hPol III in ECs resolved RPC10 in two conformational states, one with the NTD-Linker extended along the RPC1 jaw to the funnel helices, sending the distal linker to the pore entrance. There in the funnel, the C-ribbon of the CTD projects to the active center, with its catalytic residues toward the RNA 3’ end [7], similar to TFIIS in Pol II [30]. In this conformation the RPC1 clamp on the downstream dsDNA binding site was partially open. In the second conformation, the RPC10 Linker makes a U-turn and folds on itself without entering the funnel, leading the CTD to lie more peripherally in an EC that may represent a more stable processive state [7]. Stable because the downstream dsDNA binding may be more secure as the RPC1 clamp is in a closed position [7], likened to Pol II [7] and bacterial Pol elongating complexes [31,32].

Thus, different allosteric positions of the RPC1 clamp on the downstream dsDNA-binding site were found coordinated with different conformations of the CTD; the funnel-extended Linker-CTD was associated with the open clamp, whereas the U-turned Linker CTD was associated with a closed clamp [7]. Biochemical transcription reactions found that the NTD with its attached Linker was required for termination-associated Pol III recycling [18]. The NTD without a linker was active to stimulate C37-dependent termination but not C37-dependent reinitiation-recycling. Remarkably, the NTD-L could activate termination-associated recycling-reinitiation by Pol III, as could full length NTD-L-CTD containing cleavage-inactivating mutations to its (catalytic) acidic hairpin residues [18]. In addition, point mutation to two of nine invariant amino acids in the central linker greatly diminished the transcript cleavage activity of C11 consistent with the importance of the central linker for dynamic access to the funnel [18]. Finally, cleavage activity could rescue arrested Pol III including at termination, but was not required for normal/routine termination. These biochemical transcription and *in vivo* data nicely fit an advanced dynamic model of the Pol III transcription cycle that requires C11 mobility in and out of the funnel to access the active center [19].

As noted above, cryo-EM structures of oligo(dT) trapped in PTC exit tunnels have advanced understanding of Pol III termination. The studies also revealed the involvement of C37 in its reported effects on increasing termination efficiency by Pol III at suboptimal oligo(dT) length as an indirect result of impinging on and decreasinging flexibility of the exit tunnel [19,20]. Earlier data provided evidence that Pol III termination involves sensing weak (rU-dA) pairing in the RNA-DNA hybrid, and that C37 may also function in this capacity [17] suggesting distinct effects contributing to termination and reinitiation-recycling [33,34].

More recent biochemical and structural analyses led to a new model in which the exit tunnel oligo(dT) terminator trap may be likened to sudden disc brakes on the moving NT strand with enough pull on the template strand to jolt the base-paired RNA 3’ end out of the active site [19]. Offsetting the template and RNA 3’ end register relative to the active site could lead to an unproductive or arrest-like state requiring transcript cleavage activity of C11 [19], reminiscent of termination-associated arrest that requires intact C37/53 and cleavage-competent C11 to resolve [35]. Data from the new analyses were developed into a model in which Pol III termination mechanisms transition with other phases of the transcription cycle [19].

### Evolution of the eukaryotic transcript cleavage subunits/factors

Although bacterial cleavage factors GreA/B function analogously to the archaeal and eukarya counterparts, we are unaware of an evolutionary lineage connecting them. The archaeal and eukaryotic intrinsic transcript cleavage factors were examined by multiple approaches and their evolutionary relationships proposed more than ten years ago [28]. The transcript cleavage factor, TFS in the archaeal single RNA Polymerase system is the predecessor of the four major subunits/factors used by Pols I, II and III [28]. While TFS is an ancillary or transiently associating factor, Pols III, II, and I adopted different versions of stably associated or integral subunit homologs, known as Rpc11, Rpb9 and Rpa12.2 (Rpa12 or A12 hereafter), respectively (Figure 1). These consist of a Zinc-finger hairpin-ribbon as N- and C-terminal domains (also referred to as N-ribbon and C-ribbon), connected by a linker. For all of these, their N-terminal domains are anchored at the polymerase periphery while the CTDs of C11 and A12 bearing the cleavage-active catalytic amino acids, transiently accesses the active site via the pore as needed.

As Pol II is the most direct functional homolog of the archaeal Pol for general transcription [1], it is interesting that its TFS homolog Rpb9 is inactive for RNA cleavage, and Pol II instead employs TFIIS for cleavage activity (Figure 1) [28]. This may help optimize flexibility for factors that exchange on and off Pol II, as well as elongation control/regulation independent of TFIIS [see introduction in 36]. This would be consistent with the idea that Pols I and III are specialized for small subsets of genes with many fewer associated factors and one integral Rpb9 homolog whose CTD is readily active for cleavage when mobilized from a storage position in which it doesn’t interfere with elongation, into the funnel-pore as needed. Thus, evolution of C11 and A12 as integral subunits appears to have been associated with a mechanism(s) for CTD mobility to rescue active center arrest [37, and refs therein]. Nonetheless, we emphasize that the essential function of C11 is not RNA cleavage although this activity may contribute to cell growth rate and possibly other reflections of well-being [18]. The essential function of C11 appears to facilitate termination-associated reinitiation-recycling, unique to Pol III and associated with NTD-Linker mobility [18].

As detailed, specialization of Pol III likely reflects coevolution of and restructuring of its tRNA substrate genes [1,38], which contain oligo(dT) only at their 3’ end, one part of a network of mechanisms that contribute to the unique termination process [33]. Pol I is also specialized, for transcription of a single, relatively long (>10 kb) gene, arranged in head to tail tandem repeats [see 39 and refs therein]. Nonetheless, Pols I and III also share other features including that they incorporated homologs of Pol II-associated TFIIF and TFIIE components, as integral or built-in subunits [2,40-45].

While Pol III recycling requires efficient termination as well as reinitiation onto transcription initiation complexes preassembled at tRNA or other genes [1,46], Rpa12 was proposed to be involved in what may be considered to be a different type of recycling. Work by Tafur et al, suggests that Rpa12 may promote recycling of the heterodimer Rpa49/34.5 (TFIIE/F homologs) on and off Pol I, and additionally to control initiation rate [47]. Thus, the Rpa12 domain mobility may be involved in allosteric movements of other parts of the Pol I complex.

A challenge has been to identify mechanisms and test models to explain the different ability of *S. cerevisiae*, *S. pombe* and human Pols III to efficiently terminate transcription at progressively shorter oligo(dT) tracts [48]. Comparative analysis of scPol III and hPol III PTCs has allowed Girbig et al. to propose that C11/RPC10 Linker folding and Linker-CTD mobility may underly this evolutionary biology [19]. Previous studies from the Muller lab reported human-specific linker folding interactions that are now proposed to bestow RPC10 with a more readily poised position in the EC from which to access the active site, thereby contributing to higher sensitivity of hPol III to weaker/shorter oligo(rU-dA) tracts than yPol III [19].

Based on considerations above, a case can be made that C11 mobility would appear to have been important in the evolution of termination-associated reinitiation-recycling activity that is unique to Pol III. The increase in Pol III termination efficiency that accompanied eukaryotic evolution may represent another manifestation of a role of the linker in RPC10 mobility, though evidence of this if any remains to be seen.

### Potential relationship of cyto-Pol III activity in cellular innate immunity

Mammalian Pol III functions in immunity in two major branches of its evolutionary tree, as a nuclear enzyme that transcribes ncRNA genes, and as cyto-Pol III that serves as a sensor of invading viral AT-rich DNA [1]. The POLR3 initiation subunits and associated mechanisms involved in termination-reinitiation-recycling is applicable to stable transcription complexes on pre-programmed tRNA and other ncRNA genes that contribute to immune pathways [1]. The POLR3 heterotrimeric initiation subunits involved in the process of termination-associated reinitiation-recycling that involves RPC1 clamp opening and clamp closing may also be relevant to the presently unknown mechanism by which cyto-Pol III transcribes viral AT-rich DNA.

Cyto-Pol III is able to use short oligo-DNA consisting of 50 base-pairs of alternating (dT-dA) sequence as template from which to synthesize 5’-pppRNA signaling molecules. Biochemical and other evidence that such 5’-pppRNA synthesis occurs from this and other sources of AT-rich DNAs by a promoter-independent mechanism seems consistent with properties of Pol III and mechanisms utilized for DNA strand melting [44,45]. In structural studies of Pol III initiation, most DNA strand separation was attributed to actions of the built-in transcription initiation-like factors of the heterotrimeric subunits, while roles of the promoter bound TFIIIB subunits appear to be stabilization of the polymerase subunits [44,45]. In those studies, the DNA under study was considerably more GC-rich than the cyto-Pol III AT-rich substrates, the latter of which may be more prone to melting and formation of an initial transcribed complex. Indeed, short 5’-ppp-RNAs of 10-14 nucleotides in length can be sufficient to activate the RIG-I sensor [49].

## Conclusions and Perspectives

The focus of this commentary are recent studies that advance our understanding of the role of the small C11/RPC10 subunit and its associated activities in transcription by Pol III. The most abundant gene products transcribed by Pol III are the tRNAs. For this, a remarkable process of high efficiency transcription that is unique to Pol III is dependent on the RPC10/C11 subunit, and in particular its mobility within the 17-subunit enzyme, to swing in and out of the funnel to access the active center. Moreover, this occurs with apparent seamless functional connectivity between transcriptional termination and reinitiation and would appear to require the coordination of large allosteric movements of downstream and active center DNA and DNA-RNA binding sites and other domains. RPC10/C11 mobility is a critical determinant.

Key is that the C11 Linker-CTD can adopt multiple conformations, outside and inside the Pol III funnel to monitor and access the active center. Ability to enter the funnel requires that the highly conserved central linker can pass through the narrow funnel entrance. Linker conservation may also reflect ability to adopt alternative structures. A wealth of C11-Linker-CTD conformations have been observed in cryo-EM hPol III structures likley reflecting intermediates. Increases in efficiency of transcription termination by Pol III at progressively shorter oligo(dT) tracts accompanied eukaryotic evolution and was recently proposed to reflect accessibility of the RNA cleavage domain to the active center as a result of human-specific Linker folding interactions. Connecting RPC10 conformations to stimuli that induce RPC1 clamp movement on dsDNA relevant to nuclear and cyto-Pol III including in innate immune signaling should be worthy challenges.

## Figures and Tables

**Table 1:** POLR3 mutations associated with severe disease following Varicella Zoster Virus infection, primary or re-emergence.

Subunit	Mutation	Functional subcomplex	Patient	Reference
POLR3A	M307V	Core	P1	[50]
POLR3A	Q707R	Core	P2	[50]
POLR3A	R437Q	Core	P3	[50]
POLR3A	R582C	Core	P4	[51]
POLR3A	E577K	Core	P10	[52]
POLR3C	L11F	Initiation	P5	[50]
POLR3C	R438G	Initiation	P2	[50]
POLR3C	R84Q	Initiation	P3	[50]
POLR3F	R50W	Initiation	P6/P7*	[53]
POLR3E	T275M	Termination-reinitiation	P9	[51]
POLR3E	D520G	Termination-reinitiation	P5	[52]

* Identical twins with same mutations and same clinical condition.

## Abbreviations:

C11: Rpc11
C37: Rpc37
EC: Elongation Complex
NTD: N-terminal Domain
NTD-L: N-Terminal Domain-and-Linker
CTD: C-Terminal Domain
PTC: Pre-Termination Complex
Pol: RNA Polymerase
hPol III: Human RNA Polymerase III
RT: Readthrough
